# Reduced Cellular Susceptibility to *In Vitro* HIV Infection Is Associated with CD4+ T Cell Quiescence

**DOI:** 10.1371/journal.pone.0045911

**Published:** 2012-09-21

**Authors:** Catherine M. Card, W. John Rutherford, Suzie Ramdahin, Xiaojian Yao, Makobu Kimani, Charles Wachihi, Joshua Kimani, Francis A. Plummer, T. Blake Ball, Keith R. Fowke

**Affiliations:** 1 Department of Medical Microbiology, University of Manitoba, Winnipeg, Canada; 2 Department of Medical Microbiology, University of Nairobi, Nairobi, Kenya; 3 Public Health Agency of Canada, Winnipeg, Canada; 4 Department of Community Health Sciences, University of Manitoba, Winnipeg, Canada; Rush University, United States of America

## Abstract

**Background:**

HIV preferentially establishes productive infection in activated CD4+ T cells. Since proportions of activated CD4+ T cells vary between individuals, this study aimed to determine if individuals with a greater proportion of activated CD4+ T cells would be more susceptible to *in vitro* HIV infection.

**Methodology/Principal Findings:**

Unstimulated peripheral blood mononuclear cells (PBMC) from various donors were inoculated with HIV_ML1956_ *in vitro.* HIV replication was evaluated by HIV p24 ELISA of culture supernatants and intracellular staining for HIV p24, which was detected by flow cytometry. Baseline T cell phenotypes and infected cell phenotypes were also evaluated by flow cytometry. *Ex vivo* phenotyping at the time of blood draw showed that elevated T cell activation and reduced Tregs were associated with increased cellular susceptibility to *in vitro* infection. Furthermore, the infected CD4+ T cell population was enriched for activated cells.

**Conclusion/Significance:**

These data suggest that CD4+ T cell quiescence provides an environment less conducive to the establishment of HIV infection by limiting the pool of activated target cells.

## Introduction

Known risks factors for sexual transmission of HIV include high viral loads in the infected partner or concurrent sexually transmitted infections in the uninfected partner. The availability of HIV-susceptible target cells in the uninfected partner may similarly influence the probability of transmission [1]. HIV preferentially establishes productive infection in activated CD4+ T cells due its dependency on host substrates for viral entry and replication [2], [3], [4], [5], [6]. Since individuals vary in their levels of activated CD4+ T cells, we hypothesize that those who have greater numbers of activated target CD4+ T cells may have elevated susceptibility to *in vitro* HIV infection.

In HIV-infected individuals, T cell activation is considered to be a major driving force in disease progression [7], [8], [9], [10], [11], but the relationship between immune activation and HIV infection susceptibility is not well defined. This issue has recently been explored in observational studies of HIV-exposed seronegative (HESN) individuals who remain uninfected by HIV, despite multiple exposures to the virus. While no single factor accounts for resistance to infection in all cases of HESN, recent studies from the Pumwani cohort point to a role for T cell immune quiescence in protection [12], [13], [14]. T cell immune quiescence refers to a state of low baseline immune activation, which was characterized by reduced frequencies of activated CD69+ CD4+ and CD8+ T cells [12], low levels of gene transcription in CD4+ T cells [13] and whole blood [14] and reduced baseline production of cytokines by CD4+ T cells [13] in HESN. Regulatory T cells (Tregs), which are involved in suppressing immune activation, were shown to be elevated in HESN from the Pumwani cohort, and represent a potential driver of T cell immune quiescence [12]. Evidence for T cell immune quiescence has also been observed in other cohorts. Low frequencies of activated T cells have been identified in HESN men who have sex with men [15], uninfected partners of HIV-infected individuals [16], [17] and HESN CSW [18]. Reduced spontaneous lymphoproliferation has also been observed in HESN compared to healthy control groups [15], [19].

In the present study, we sought to characterize T cell phenotypes before and after *in vitro* HIV infection and examined the relationship between infection susceptibility and target cell activation. We show that elevated cellular susceptibility to infection is associated with high levels of T cell activation *ex vivo* and that HIV preferentially targets activated CD4+ T cells.

## Results

### PBMC from Select Individuals Demonstrate Relative Resistance to HIV Infection in vitro

Unstimulated PBMC from 21 HIV-uninfected study participants from the Pumwani cohort were infected with HIV_ML1956_ at a MOI of 0.1 in six replicate wells. Virus levels were quantified in supernatants collected on day 9 post-inoculation.

**Figure 1:**
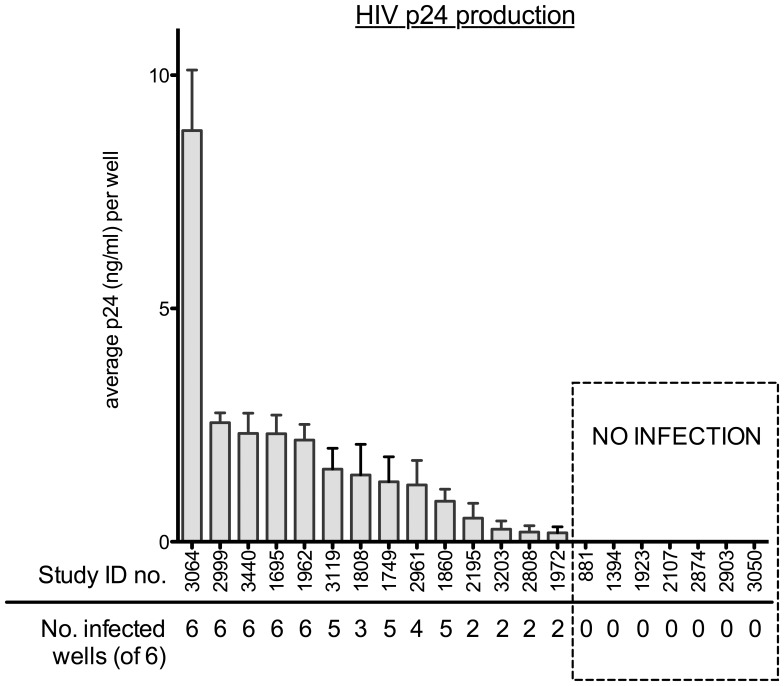
Infection of unstimulated PBMC inoculated with HIV_ML1956_. Graph shows average level of HIV infection of replicate wells as determined by HIV p24 ELISA. Individual patients are represented by their study ID numbers. The number of replicate wells demonstrating productive infection (out of 6) is indicated below the patient study ID numbers.

The level of viral production varied between individuals. Fourteen of 21 individuals demonstrated productive infection in at least two replicate wells. Five individuals demonstrated productive infection in all six replicate wells and had higher average levels of virus production per well compared to the other participants. At the other end of the spectrum, seven individuals had no detectable p24 levels in any of the replicate wells (Figure 1).

Since study subjects were high-risk HIV-uninfected participants enrolled in the Pumwani commercial sex worker cohort, this study population included individuals who are HESN. In this cohort, we have defined an extreme phenotype of HESN, in which individuals are considered HESN if they remained HIV-uninfected for greater than 7 years of follow-up in the Pumwani cohort and were active in sex work during that time. Therefore, the proportion of individuals meeting these criteria for HESN was compared between those individuals with detectable viral replication and those with no detectable viral replication. No difference was detected in the proportion of HESN among individuals who did have detectable levels of viral replication (7/14) and those without detectable levels of viral replication (4/7) (p = 1.0). Age was also not significantly different between groups (p = 0.35).

### High Levels of T Cell Activation and Reduced Tregs at Baseline are Associated with Elevated Susceptibility to HIV Infection in vitro

**Figure 2:**
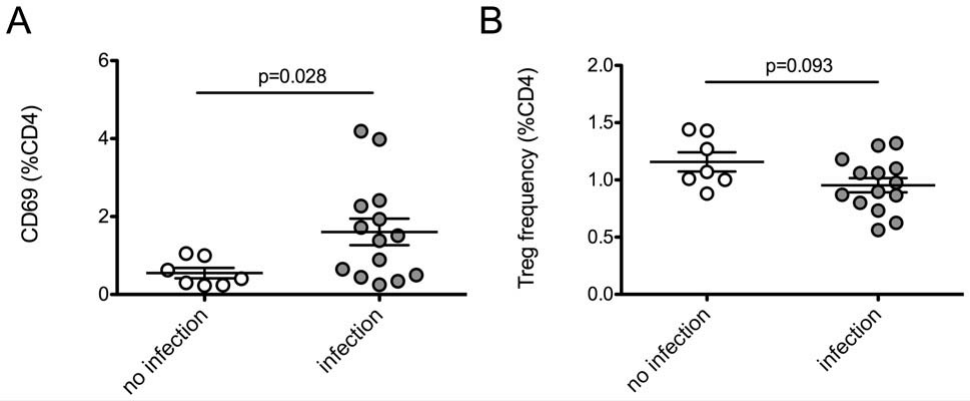
T cell phenotypes of unstimulated PBMC with differential susceptibility to *in vitro* infection with HIV_ML1956_. PBMC were immunophenotyped *ex vivo* to assess T cell activation and Tregs prior to inoculation with HIV_ML1956_ *in vitro*. PBMC with no detectable infection (n = 7) were compared to those demonstrating productive infection (n = 14) at 9 days post-inoculation. (A) PBMC with undetectable levels of virus production by day 9 post-inoculation had lower frequencies of CD4+ CD69+ T cells. (B) PBMC with undetectable levels of virus production by day 9 post-inoculation had higher frequencies of Tregs (defined as CD25^hi^ FOXP3+), but the difference was not statistically significant. P-values were calculated using the Mann-Whitney *U* test.

PBMC were phenotyped *ex vivo* (day 0) to address the roles of baseline T cell activation and Tregs in cellular susceptibility to infection. Baseline T cell phenotypes were compared between the 14 individuals who demonstrated viral replication and the seven individuals with no detectable viral replication (Table 1). Individuals with detectable levels of virus production had significantly higher baseline levels of CD4+ CD69+ T cells (p = 0.028). In addition, a trend was observed in which the presence of infection was associated with a modest reduction in Treg frequency (p = 0.093) (Figure 2).

**Table 1:** Effect of *ex vivo* T cell phenotype on susceptibility of unstimulated PBMC to infection with HIV_ML1956_ virus at a MOI of 0.1.

T cell phenotype	No Detectable Virus (n = 7)	Virus Detected (n = 14)	p-value^a^
Relative Expression^b^			
** CD4+ CD69+**	**0.41 (0.24–1.00)**	**1.45 (0.48–2.31)**	**0.028**
CD4+ CD38+	38.8 (23.8–49.6)	42.3 (34.9–46.2)	0.628
CD4+ HLA DR+	1.56 (0.80–2.32)	1.65 (1.24–2.48)	0.479
CD4+ CD38+/HLA DR+	0.75 (0.28–1.20)	0.73 (0.61–1.24)	0.737
CD4+ CCR5+	0.70 (0.46–1.56)	0.99 (0.63–1.35)	0.628
CD4+ CXCR4+	73.6 (35.2–86.1)	54.0 (40.8–84.0)	0.970
CD8+ CD69+	1.06 (0.53–1.88)	1.91 (0.67–4.59)	0.192
CD8+ CD38+	47.2 (41.1–48.2)	44.9 (43.3–46.8)	0.852
CD8+ HLA DR+	4.90 (2.92–8.60)	4.52 (3.56–5.28)	0.794
CD8+ CD38+/HLA DR+	2.81 (1.58–4.50)	2.44 (1.92–2.74)	0.682
** Tregs (CD25+ FOXP3+)**	**1.07 (1.00–1.43)**	**0.94 (0.78–1.12)**	**0.093**
Treg/CTLA-4+	23.2 (11.1–26.4)	22.0 (18.9–27.7)	0.682
Estimated number of cells^c^			
** CD4+ CD69+**	**314.7 (156.5–432.5)**	**677.6 (311.4–1150)**	**0.093**
CD4+ CD38+	19195 (15565–35354)	24400 (14117–29307)	0.970
CD4+ HLA DR+	804.6 (520.9–1212)	1020 (663.4–1335)	0.737
CD4+ CD38+/HLA DR+	476.1 (182.6–585.1)	476.1 (281.0–688.7)	0.794
CD4+ CCR5+	452.8 (206.1–1195)	482.4 (344.7–881.0)	0.970
CD4+ CXCR4+	47987 (15983–59581)	31633 (18104–51924)	0.576
** CD8+ CD69+**	**210.9 (109.2–332.8)**	**379.3 (183.3–1013)**	**0.093**
CD8+ CD38+	8514 (7642–12602)	10586 (7726–13124)	0.433
CD8+ HLA DR+	779.6 (499.8–2236)	1139 (798.6–1341)	0.852
CD8+ CD38+/HLA DR+	563.4 (286.6–1160)	615.7 (450.7–743.0)	0.970
Tregs (CD25+ FOXP3+)	740.4 (393.4–972.8)	537.6 (347.2–717.4)	0.109
Treg/CTLA-4+	171.8 (42.66–285.3)	107.0 (62.77–222.3)	0.433

a p-values calculated using non-parametric Mann-Whitney *U* test. Parameters that demonstrated statistically significant (p<0.05) or trending (p<0.10) p-values are highlighted in bold text.

b Relative expression of phenotypic markers is listed as median of percent positive (+) cells. Interquartile range is shown in parentheses.

c Estimated number of cells was calculated from flow cytometry proportions based on seeding 10^5^ cells per well. Interquartile range is shown in parentheses.

Since comparing proportions of activated cells between groups does not account for differences in the size of CD4+ or CD8+ T cell populations, the estimated number of cells of each phenotype were calculated based on the percentages observed by flow cytometry. These calculated T cell counts were compared between the 14 individuals who demonstrated viral replication and the seven individuals with no detectable viral replication (Table 1). Consistent with the differences in percentages of CD4+ CD69+ T cells between groups, a trend was observed in which individuals with detectable levels of virus production had higher baseline levels of total CD4+ CD69+ T cells (p = 0.093). Although the calculated number of Tregs was lower in individuals with detectable viral replication, this difference was not statistically significant. Also of note, the estimated count of CD8+ CD69+ T cells was higher in individuals demonstrating detectable viral replication, consistent with an activated T cell phenotype in these subjects.

### Activated CD4+ T Cells are Enriched in the HIV p24+ Cell Population

**Figure 3:**
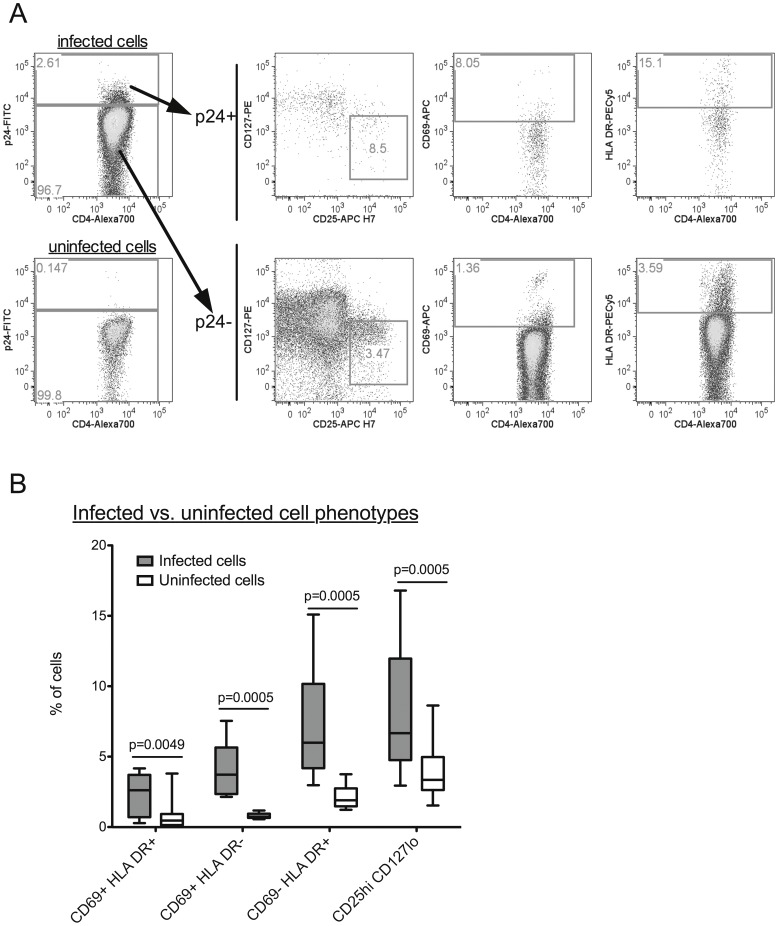
Phenotypes of HIV-infected unstimulated CD4+ T cells. (A) CD4+ T cells were evaluated for infection by intracellular staining for HIV p24. Cells were gated on singlets, lymphocytes and live cells before discriminating CD4+ T cells. Infected (p24+) and uninfected cells (p24-) were phenotyped for markers of Tregs (defined as CD25^hi^, CD127^lo^) and T cell activation (CD69, HLA DR). (B) Comparison of T cell phenotypes between infected and uninfected CD4+ T cells (n = 12). Higher frequencies of infected cells were activated (CD69+ HLA DR+, CD69+ HLA DR- or CD69- HLA DR+) or demonstrated a Treg phenotype (CD25^hi^ CD127^lo^) compared to uninfected cells. P-values were calculated using the Wilcoxon signed-rank test for matched pairs.

When infection cultures were terminated, cells were harvested and stained for intracellular HIV p24 and markers of T cell activation and Tregs (Figure 3A). T cell phenotypes were compared between infected (HIV p24+) and uninfected (HIV p24-) CD4+ T cells from infection cultures (n = 12). A higher proportion of infected cells were CD69+ HLA DR+ (p = 0.005), CD69+ HLA DR- (p = 0.0005) or CD69- HLA DR+ (p = 0.0005) compared to uninfected cells (Figure 3B). In addition to CD69 and HLA DR, expression of CD25 and CD127 was evaluated on infected cells. A greater proportion of infected cells were CD25^hi^ CD127^lo^ compared to uninfected cells (p = 0.0005) (Figure 3B). This is consistent with a Treg phenotype, but these markers may also reflect the phenotype of activated conventional T cells.

## Discussion

HIV preferentially establishes productive infection in activated T cells. While it is established that immune activation is a major driving factor in HIV disease progression, its relevance in susceptibility to infection is not well understood. We hypothesized that cells from individuals with high levels of baseline immune activation would be more susceptible to *in vitro* HIV infection. As such, we assessed the infectability of unstimulated PBMC from 21 HIV-uninfected women and addressed the role of target cell activation on susceptibility to infection.

Of the 21 samples exposed to HIV_ML1956_ at an MOI of 0.1, one third showed no evidence of HIV replication following 9 days of culture. However, lack of detectable viral replication does not imply absolute resistance to infection. The assay used in the present study does not rule out the presence of low levels of viral replication or latent infection.

Baseline T cell phenotypes were analyzed in unstimulated PBMC prior to inoculation with virus, demonstrating that individuals with detectable levels of HIV replication by day 9 post-infection had significantly higher levels of CD4+ CD69+ T cells and a trend toward lower levels of Tregs compared to those with no detectable viral replication by day 9 (Figure 2). This *in vitro* data supports our previous observation of low CD4+ CD69+ T cells and elevated Tregs in *ex vivo* PBMC from HESN women compared to susceptible controls [12].

There was no association between in vitro HIV susceptibility and expression of the chronic activation markers HLA DR or CD38 (Table 1). This suggests that increased cellular susceptibility to *in vitro* infection is largely a consequence of activation at the earliest stages of the cell cycle. Future studies should include additional markers of cell cycle entry such as Ki67 to confirm this observation.

High levels of T cell activation may promote HIV infection by increasing the availability of activated target cells. HIV can enter quiescent CD4+ T cells [20], [21], [22], [23], [24], but the processes of reverse transcription and integration are inefficient and often result in abortive infections [20], [21], [23]. CD69 is expressed early in the cell cycle, indicating that CD69- cells are quiescent, and do not effectively support HIV replication [25], [26]. As such, the association between lack of detectable viral replication on day 9 post-inoculation and reduced baseline frequencies of CD4+ CD69+ T cells supports the hypothesis that T cell immune quiescence can limit infection by reducing the pool of activated HIV target cells. Tregs suppress T cell activation and responses, so elevated levels of Tregs may protect against HIV infection by limiting activation of target CD4+ T cells. Additionally, Tregs can directly suppress HIV infection through a cAMP-dependent mechanism [27], [28]. HIV replication is negatively regulated by cAMP due to inhibition of HIV DNA nuclear import, integration and transcription [29], [30], and blockage of HIV LTR activation by NF-κB [31]. Further studies involving Treg depletion experiments will be needed to ascertain the role of Tregs in modulating susceptibility of unstimulated PBMC to HIV infection *in vitro*.

To characterize the activation status of cells that did support HIV replication, phenotypes of infected cells were analyzed by flow cytometry. Consistent with the preferential infection of activated CD4+ T cells by HIV, the infected (HIV p24+) CD4+ T cell population contained a significantly higher proportion of activated T cells than the uninfected (HIV p24-) CD4+ T cell population. Tregs were also significantly enriched within the HIV p24+ CD4+ T cell population. However, it should be noted that in the present study, Tregs were defined by expression of high levels of CD25 and low levels of CD127/IL-7Rα, and not by FOXP3, due to complications with co-staining of the intracellular markers p24 and FOXP3. Since activated cells upregulate CD25 and downregulate CD127, the Treg populations evaluated here may not be pure, and potentially represent some activated cells. As discussed above, Tregs suppress immune activation so they may play a beneficial role in preventing infection. However, Tregs can also be infected with HIV [32], [33], [34], suggesting that elevated Tregs may actually provide additional target cells for HIV infection.

To summarize, increased cellular susceptibility to *in vitro* HIV infection was associated with elevated activated CD4+ T cells and a trend toward reduced Tregs *ex vivo*. Furthermore, viral production was preferentially established in activated CD4+ T cells. These data are supported by findings in non-human primate models of SIV transmission, in which acute SIV infection is established in resting cells, but viral propagation and dissemination are fuelled by activated target cells present at sites of viral replication [2], [3], [35], [36]. Interestingly, use of the anti-inflammatory compound glycerol monolaurate blunted the inflammatory response and prevented infiltration of activated target cells, and resulted in protection from SIV infection [37].

There are several limitations to this study. First, the study subjects are high-risk HIV-uninfected women from the Pumwani cohort, and a proportion of these individuals are HESN. Lack of virus production was not restricted to HESN, and individuals with detectable levels of productive infection did not differ from those with no detectable productive infection in the proportion of participants who were HESN. However, it is likely that some of these subjects have additional mechanisms of resistance to infection. Future studies should include a low-risk HIV-uninfected control group and evaluate the effect of HESN status on susceptibility to *in vitro* infection. Second, the limited sensitivity of the HIV p24 ELISA and delayed kinetics of unstimulated infections required prolonged maintenance of cells in culture. Cultures with undetectable infection may have been latently infected or had low levels of viral replication. In addition, changes in the phenotype of the population of cells can be expected to occur over time. As such, future studies should employ more sensitive infection readouts and earlier time points.

The data presented here, along with observational studies in HESN [12], [13], [14], [15], [16], [17], [18], [19] and animal models [2], [3], [35], [36], [37], demonstrate that T cell immune quiescence is associated with resistance to HIV infection. The T cell immune quiescence model suggests that mechanisms of reducing inflammation and target cell activation should be considered during future HIV vaccine and microbicide development.

## Materials and Methods

### Study Subjects and Ethics Statement

The Research Ethics Boards at the University of Manitoba and Kenyatta National Hospital approved the study protocols. Study subjects were high-risk HIV-uninfected women enrolled in the Pumwani Commercial Sex Worker Cohort based in Nairobi, Kenya. Subjects were considered HESN if they remained HIV-uninfected for greater than 7 years of follow-up in the Pumwani cohort and were active in sex work during that time. Written informed consent was obtained from all study participants.

### Virus Strain

The HIV-1 strain that was used for the *in vitro* infections was HIV_ML1956_, a primary isolate from a HIV-infected participant of the Pumwani cohort. HIV_ML1956_ was originally derived by primary co-culture with PHA-stimulated PBMC [38]. Briefly, PBMC from HIV-uninfected donors were stimulated with PHA for 3 days then co-cultured with PHA-stimulated PBMC from the HIV-infected patient. Co-cultures were maintained in RPMI containing 10% FCS, 1% Penicillin/Streptomycin/Fungizone and 100 IU IL-2, and were supplemented periodically with fresh PHA-stimulated feeder cells. Virus production was monitored in culture supernatant by HIV p24 ELISA. The supernatant was harvested at peak p24 production, and the virus was collected and stored at −135°C for future studies.

Full-length HIV proviral sequencing performed on the HIV_ML1956_ isolate revealed that it is a recombinant of clades A2, C and D, consistent with a high proportion of circulating recombinant viruses in the cohort [39].

### Infection of PBMC in vitro

Unstimulated PBMC (10^5^ cells/well in 96 well microplates) from HIV-uninfected women (n = 21) were inoculated with HIV_ML1956_ at a multiplicity of infection (MOI) of 0.1 in six replicate wells. Cells were incubated with virus for 4 hours in a total volume of 200 µl/well. The cells were then washed twice by centrifugation at 1200rpm for 5 minutes, and 150 µl of media was removed from the wells and replaced with fresh media after each wash. Cultures were maintained in RPMI containing 10% FCS, 1% Penicillin/Streptomycin/Fungizone and 10 IU IL-2. On day 3 post-inoculation, 100 µl of supernatant was removed from each well and replaced with 100 µl of fresh media. On days 9 and 16 post-inoculation of unstimulated cells, 100 µl of supernatant was harvested from each well and treated with 10 µl of 10% Triton-X-100 then frozen at −80°C for later HIV p24 determinations. Infection cultures were then terminated and cells were harvested for evaluation of infected cell phenotypes by flow cytometry.

### HIV-1 Quantification in Supernatants

HIV p24 was measured in supernatants from infection cultures using a sandwich enzyme-linked immunosorbent assay (ELISA). ELISA plates were coated with anti-HIV p24 monoclonal IgG1 purified from supernatants of HIV-1 p24 hybridoma cells (183-H12-5C, NIH AIDS Research and Reference Reagent Program, Division of AIDS, NIAID, NIH). Frozen supernatants from infection cultures were thawed and 75 µl was transferred to ELISA plates. Detection of HIV p24 in supernatants was done using rabbit polyclonal anti-p24 detection antibody (Advanced Biotechnologies), followed by a biotin anti-rabbit goat IgG secondary antibody (Sigma). Streptavidin horseradish peroxidase (Invitrogen) and tetramethyl benzidine substrate solution (Invitrogen) were used detect binding of the secondary antibody. When plates were developed, 3% HCl was added to each well to stop the reaction. Optical density of wells was read at OD_450nm_ on a SpectraMax Plus spectrophotometer (Molecular Devices). Data were acquired and analyzed using SoftMax Pro software, version 3.1.2 (Molecular Devices). The ELISA detected HIV p24 concentrations ranging from 280 to 0.273 ng/ml.

### Flow Cytometry

PBMC were immunophenotyped to assess *ex vivo* T cell phenotypes at baseline (i.e. prior to infection). To assess T cell activation and HIV coreceptor expression, cells were stained with anti-CD69-FITC, anti-CD38-PECy5, anti-HLADR-APCCy7, anti-CCR5-PE, anti-CXCR4-PECy7, anti-CD4-AlexaFluor700, anti-CD3-Pacific Blue (all BD Biosciences), anti-CD8-PETR (Invitrogen) and Live/Dead Aqua dye (Invitrogen). To evaluate Tregs, cells were stained with anti-CD25-PE, anti-CTLA4-PECy5, anti-CD4-AlexaFluor700, anti-CD3-Pacific Blue (all BD Biosciences), anti-FOXP3-APC (clone PCH101, eBioscience) and Live/Dead Aqua dye (Invitrogen). FOXP3 staining was performed using the FOXP3 Staining Buffer Set (eBioscience). Tregs were defined as CD4+ T cells co-expressing high levels of CD25 and FOXP3. The total number of CD4+ or CD8+ T cells per well was calculated from flow cytometry percentages (i.e. CD4+ or CD8+ T cells as percent of total events) based on seeding 10^5^ cells per well. Estimated number of cells of each phenotype was further calculated using percentages obtained by flow cytometry.

Cells collected from infection cultures were stained with anti-HLADR-PECy5, anti-CD69-APC, anti-CD127-PE, anti-CD25-APCCy7, anti-CD4-AlexaFluor700 (all BD Biosciences), anti-CD3-PETR (Invitrogen), anti-p24-FITC (KC57, Beckman Coulter) and Live/Dead Aqua dye (Invitrogen) to evaluate phenotypes of infected cells. Due to complications of co-staining HIV p24 and FOXP3, Tregs were defined by high expression of CD25 and low expression of CD127 for the analysis of infected cell phenotypes.

Stained samples were acquired on a LSRII flow cytometer (BD Biosciences). Samples were suspended in 250 µl of fixation buffer (BD Biosciences) prior to acquisition. When acquiring data, 50,000–100,000 lymphocyte events were acquired for each sample. Analysis of flow cytometry data was performed using FlowJo software, version 9.3.1 (Tree Star Inc., Ashland, OR).

### Statistical Analysis

Comparison of HESN status between groups of individuals was performed using Fisher’s Exact Test. Comparisons of baseline phenotypes between groups of individuals were performed using non-parametric Mann-Whitney *U* tests. Comparisons of T cell phenotypes between infected and uninfected cells were performed using the Wilcoxon signed-rank test for matched pairs. Differences were considered to be statistically significant at p<0.05. All statistical analyses were performed using GraphPad Prism for Mac OS X, version 5.0a (GraphPad software, La Jolla, CA).

## References

[1] McKinnon LR, Kaul R (2012) Quality and quantity: mucosal CD4+ T cells and HIV susceptibility. Current Opinion in HIV and AIDS 7: 195–202. 22314505 10.1097/COH.0b013e3283504941

[2] Zhang Z, Schuler T, Zupancic M, Wietgrefe S, Staskus KA, et al (1999) Sexual transmission and propagation of SIV and HIV in resting and activated CD4+ T cells. Science 286: 1353–1357. 10558989 10.1126/science.286.5443.1353

[3] Zhang Z-Q, Wietgrefe SW, Li Q, Shore MD, Duan L, et al (2004) Roles of substrate availability and infection of resting and activated CD4+ T cells in transmission and acute simian immunodeficiency virus infection. Proc Natl Acad Sci U S A 101: 5640–5645. 15064398 10.1073/pnas.0308425101 PMC397458

[4] Brass AL, Dykxhoorn DM, Benita Y, Yan N, Engelman A, et al (2008) Identification of Host Proteins Required for HIV Infection Through a Functional Genomic Screen. Science 319: 921–926. 18187620 10.1126/science.1152725

[5] König R, Zhou Y, Elleder D, Diamond TL, Bonamy GMC, et al (2008) Global Analysis of Host-Pathogen Interactions that Regulate Early-Stage HIV-1 Replication. Cell 135: 49–60. 18854154 10.1016/j.cell.2008.07.032 PMC2628946

[6] Zhou H, Xu M, Huang Q, Gates AT, Zhang XD, et al (2008) Genome-Scale RNAi Screen for Host Factors Required for HIV Replication. Cell Host and Microbe 4: 495–504. 18976975 10.1016/j.chom.2008.10.004

[7] Deeks SG (2004) Immune activation set point during early HIV infection predicts subsequent CD4+ T-cell changes independent of viral load. Blood 104: 942–947. 15117761 10.1182/blood-2003-09-3333

[8] Giorgi J, Hultin L, McKeating J (1999) Shorter survival in advanced human immunodeficiency virus type 1 infection is more closely associated with T lymphocyte activation than with plasma virus burden or virus chemokine coreceptor usage. J Infect Dis 179: 859–870. 10068581 10.1086/314660

[9] Giorgi JV, Lyles RH, Matud JL, Yamashita TE, Mellors JW, et al (2002) Predictive value of immunologic and virologic markers after long or short duration of HIV-1 infection. J Acquir Immune Defic Syndr 29: 346–355. 11917238 10.1097/00126334-200204010-00004

[10] Hunt PW, Brenchley J, Sinclair E, McCune JM, Roland M, et al (2008) Relationship between T cell activation and CD4+ T cell count in HIV-seropositive individuals with undetectable plasma HIV RNA levels in the absence of therapy. J Infect Dis 197: 126–133. 18171295 10.1086/524143 PMC3466592

[11] Liu Z, Cumberland WG, Hultin LE, Prince HE, Detels R, et al (1997) Elevated CD38 antigen expression on CD8+ T cells is a stronger marker for the risk of chronic HIV disease progression to AIDS and death in the Multicenter AIDS Cohort Study than CD4+ cell count, soluble immune activation markers, or combinations of HLA-DR and CD38 expression. J Acquir Immune Defic Syndr 16: 83–92. 10.1097/00042560-199710010-00003 9358102

[12] Card CM, McLaren PJ, Wachihi C, Kimani J, Plummer FA, et al (2009) Decreased immune activation in resistance to HIV-1 infection is associated with an elevated frequency of CD4(+)CD25(+)FOXP3(+) regulatory T cells. J Infect Dis 199: 1318–1322. 19301980 10.1086/597801

[13] McLaren P, Ball T, Wachihi C, Jaoko W, Kelvin D, et al (2010) HIV-Exposed Seronegative Commercial Sex Workers Show a Quiescent Phenotype in the CD4+ T Cell Compartment and Reduced Expression of HIV-Dependent Host Factors. J Infect Dis 202: S339–S344. 20887221 10.1086/655968

[14] Songok EM, Luo M, Liang B, McLaren P, Kaefer N, et al (2012) Microarray Analysis of HIV Resistant Female Sex Workers Reveal a Gene Expression Signature Pattern Reminiscent of a Lowered Immune Activation State. PLoS ONE 7: e30048. 22291902 10.1371/journal.pone.0030048 PMC3266890

[15] Koning FA, Otto SA, Hazenberg MD, Dekker L, Prins M, et al (2005) Low-level CD4+ T cell activation is associated with low susceptibility to HIV-1 infection. J Immunol 175: 6117–6122. 16237108 10.4049/jimmunol.175.9.6117

[16] Camara M, Dieye TN, Seydi M, Diallo AA, Fall M, et al (2010) Low-level CD4+ T cell activation in HIV-exposed seronegative subjects: influence of gender and condom use. J Infect Dis 201: 835–842. 20136413 10.1086/651000

[17] Begaud E, Chartier L, Marechal V, Ipero J, Leal J, et al (2006) Reduced CD4 T cell activation and in vitro susceptibility to HIV-1 infection in exposed uninfected Central Africans. Retrovirology 3: 35. 16792805 10.1186/1742-4690-3-35 PMC1524799

[18] Jennes W, Evertse D, Borget M-Y, Vuylsteke B, Maurice C, et al (2006) Suppressed cellular alloimmune responses in HIV-exposed seronegative female sex workers. Clin Exp Immunol 143: 435–444. 16487242 10.1111/j.1365-2249.2006.03017.x PMC1809613

[19] Salkowitz JR, Purvis SF, Meyerson H, Zimmerman P, O’Brien TR, et al (2001) Characterization of high-risk HIV-1 seronegative hemophiliacs. Clin Immunol 98: 200–211. 11161976 10.1006/clim.2000.4969

[20] Zack JA, Arrigo SJ, Weitsman SR, Go AS, Haislip A, et al (1990) HIV-1 entry into quiescent primary lymphocytes: molecular analysis reveals a labile, latent viral structure. Cell 61: 213–222. 2331748 10.1016/0092-8674(90)90802-l

[21] Spina CA, Guatelli JC, Richman DD (1995) Establishment of a stable, inducible form of human immunodeficiency virus type 1 DNA in quiescent CD4 lymphocytes in vitro. J Virol 69: 2977–2988. 7707524 10.1128/jvi.69.5.2977-2988.1995 PMC188997

[22] Tang S, Patterson B, Levy JA (1995) Highly purified quiescent human peripheral blood CD4+ T cells are infectible by human immunodeficiency virus but do not release virus after activation. J Virol 69: 5659–5665. 7637012 10.1128/jvi.69.9.5659-5665.1995 PMC189423

[23] Zack JA, Haislip AM, Krogstad P, Chen IS (1992) Incompletely reverse-transcribed human immunodeficiency virus type 1 genomes in quiescent cells can function as intermediates in the retroviral life cycle. J Virol 66: 1717–1725. 1371173 10.1128/jvi.66.3.1717-1725.1992 PMC240919

[24] Chun TW, Finzi D, Margolick J, Chadwick K, Schwartz D, et al (1995) In vivo fate of HIV-1-infected T cells: quantitative analysis of the transition to stable latency. Nat Med 1: 1284–1290. 7489410 10.1038/nm1295-1284

[25] Vatakis DN, Bristol G, Wilkinson TA, Chow SA, Zack JA (2007) Immediate activation fails to rescue efficient human immunodeficiency virus replication in quiescent CD4+ T cells. J Virol 81: 3574–3582. 17229711 10.1128/JVI.02569-06 PMC1866069

[26] Korin YD, Zack JA (1998) Progression to the G1b phase of the cell cycle is required for completion of human immunodeficiency virus type 1 reverse transcription in T cells. J Virol 72: 3161–3168. 9525642 10.1128/jvi.72.4.3161-3168.1998 PMC109773

[27] Moreno-Fernandez ME, Rueda CM, Rusie LK, Chougnet CA (2011) Regulatory T cells control HIV replication in activated T cells through a cAMP-dependent mechanism. Blood 117: 5372–5380. 21436067 10.1182/blood-2010-12-323162 PMC3109711

[28] Moreno-Fernandez ME, Rueda CM, Velilla PA, Rugeles MT, Chougnet CA (2011) cAMP During HIV Infection: Friend or Foe? AIDS research and human retroviruses. 10.1089/aid.2011.0265 PMC3251837 21916808

[29] Navarro J, Punzón C, Jiménez JL, Fernández-Cruz E, Pizarro A, et al (1998) Inhibition of phosphodiesterase type IV suppresses human immunodeficiency virus type 1 replication and cytokine production in primary T cells: involvement of NF-kappaB and NFAT. J Virol 72: 4712–4720. 9573235 10.1128/jvi.72.6.4712-4720.1998 PMC109998

[30] Sun Y, Li L, Lau F, Beavo JA, Clark EA (2000) Infection of CD4+ memory T cells by HIV-1 requires expression of phosphodiesterase 4. J Immunol 165: 1755–1761. 10925252 10.4049/jimmunol.165.4.1755

[31] Banas B, Eberle J, Banas B, Schlöndorff D, Luckow B (2001) Modulation of HIV-1 enhancer activity and virus production by cAMP. FEBS letters 509: 207–212. 11741590 10.1016/s0014-5793(01)03182-9

[32] Oswald-Richter K, Grill SM, Shariat N, Leelawong M, Sundrud MS, et al (2004) HIV infection of naturally occurring and genetically reprogrammed human regulatory T-cells. PLoS Biol 2: E198. 15252446 10.1371/journal.pbio.0020198 PMC449855

[33] Antons AK, Wang R, Oswald-Richter K, Tseng M, Arendt CW, et al (2008) Naive precursors of human regulatory T cells require FoxP3 for suppression and are susceptible to HIV infection. J Immunol 180: 764–773. 18178814 10.4049/jimmunol.180.2.764

[34] Moreno-Fernandez ME, Zapata W, Blackard JT, Franchini G, Chougnet CA (2009) Human regulatory T cells are targets for human immunodeficiency Virus (HIV) infection, and their susceptibility differs depending on the HIV type 1 strain. J Virol 83: 12925–12933. 19828616 10.1128/JVI.01352-09 PMC2786841

[35] Miller CJ, Li Q, Abel K, Kim E-Y, Ma ZM, et al (2005) Propagation and dissemination of infection after vaginal transmission of simian immunodeficiency virus. J Virol 79: 9217–9227. 15994816 10.1128/JVI.79.14.9217-9227.2005 PMC1168785

[36] Haase AT (2010) Targeting early infection to prevent HIV-1 mucosal transmission. Nature 464: 217–223. 20220840 10.1038/nature08757

[37] Li Q, Estes JD, Schlievert PM, Duan L, Brosnahan AJ, et al (2009) Glycerol monolaurate prevents mucosal SIV transmission. Nature 458: 1034–1038. 19262509 10.1038/nature07831 PMC2785041

[38] Lane JR (1999) Isolation and Expansion of HIV from Cells and Body Fluids by Coculture. Methods Mol Med 17: 3–10. 21380650 10.1385/0-89603-369-4:3

[39] Land AM, Ball TB, Luo M, Rutherford J, Sarna C, et al (2008) Full-length HIV type 1 proviral sequencing of 10 highly exposed women from Nairobi, Kenya reveals a high proportion of intersubtype recombinants. AIDS Res Hum Retroviruses 24: 865–872. 18544023 10.1089/aid.2007.0200

